# Human epidermal growth factor receptor-2 and endocrine resistance in hormone-dependent breast cancer

**DOI:** 10.1530/ERC-21-0293

**Published:** 2022-05-25

**Authors:** Anastasia Alataki, Mitch Dowsett

**Affiliations:** 1 Ralph Lauren Centre for Breast Cancer Research, Royal Marsden Hospital and The Institute of Cancer Research, London, UK; 2 The Breast Cancer Now Toby Robins Research Centre, The Institute of Cancer Research, London, UK

**Keywords:** breast cancer, oestrogen receptor, human epidermal growth factor receptor-2, resistance, endocrine therapies

## Abstract

Endocrine therapies are the main treatment strategies for the clinical management of hormone-dependent breast cancer. Despite prolonged time to recurrence in the adjuvant setting and the initial clinical responses in the metastatic setting, many patients eventually encounter tumour relapse due to acquired resistance to these agents. Other patients experience a lack of tumour regression at the beginning of treatment indicating *de novo* resistance that significantly limits its efficacy in the clinic. There is compelling evidence that human epidermal growth factor receptor-2 (HER2) overexpression contributes to resistance to endocrine therapies in oestrogen receptor-positive (ER+) breast cancer. ER+/HER2+ tumours comprise about 10% of all breast cancer cases and about 60% of the whole set of HER2+ tumours. Most patients with primary ER+/HER2+ disease will receive antibody-based HER2-targeted therapy, but this is generally for no more than one year while endocrine treatment is usually for at least 5 years. A number of HER2-kinase inhibitors are also now in clinical use or in clinical trials, and the interaction of these with endocrine treatment may differ from that of antibody treatment. In this review article, we aim to summarise knowledge on molecular mechanisms of breast cancer resistance to endocrine therapies attributable to the impact of HER2 signalling on endocrine sensitivity, to discuss data from clinical trials addressing the role of HER2 in the development of endocrine resistance in the metastatic, neoadjuvant and adjuvant settings and to explore rational new therapeutic strategies.

## Introduction

Breast cancer development and progression are significantly affected by signalling pathways involving oestrogen receptor (ER) and growth factor receptors (Arpino *et al.* 2008). Over 80% of all breast cancer cases are deemed ER-positive and/or progesterone receptor (PgR)-positive (Dodson *et al.* 2018), and oestrogen is the primary growth stimulant of these tumours (Dixon 2014). Hormone receptor (HR) status is measured in all primary breast cancers and is used as a predictive marker for selecting patients, who are more likely to benefit from hormonal therapy strategies whether this will be in the early or the metastatic context (Ring & Dowsett 2003). Among these therapies are the selective ER modulator, tamoxifen, which inhibits tumour growth by binding and blocking ER (EBCTCG 1998) and aromatase inhibitors (AIs) that inhibit the enzyme that converts androgens into oestrogen (Gradishar 2004). When given as adjuvant therapy to postmenopausal women, 5 years’ tamoxifen or AI reduce the risk of patients dying from their breast cancer by about 30 or 40%, respectively (EBCTCG 2015).

**Figure 1:**
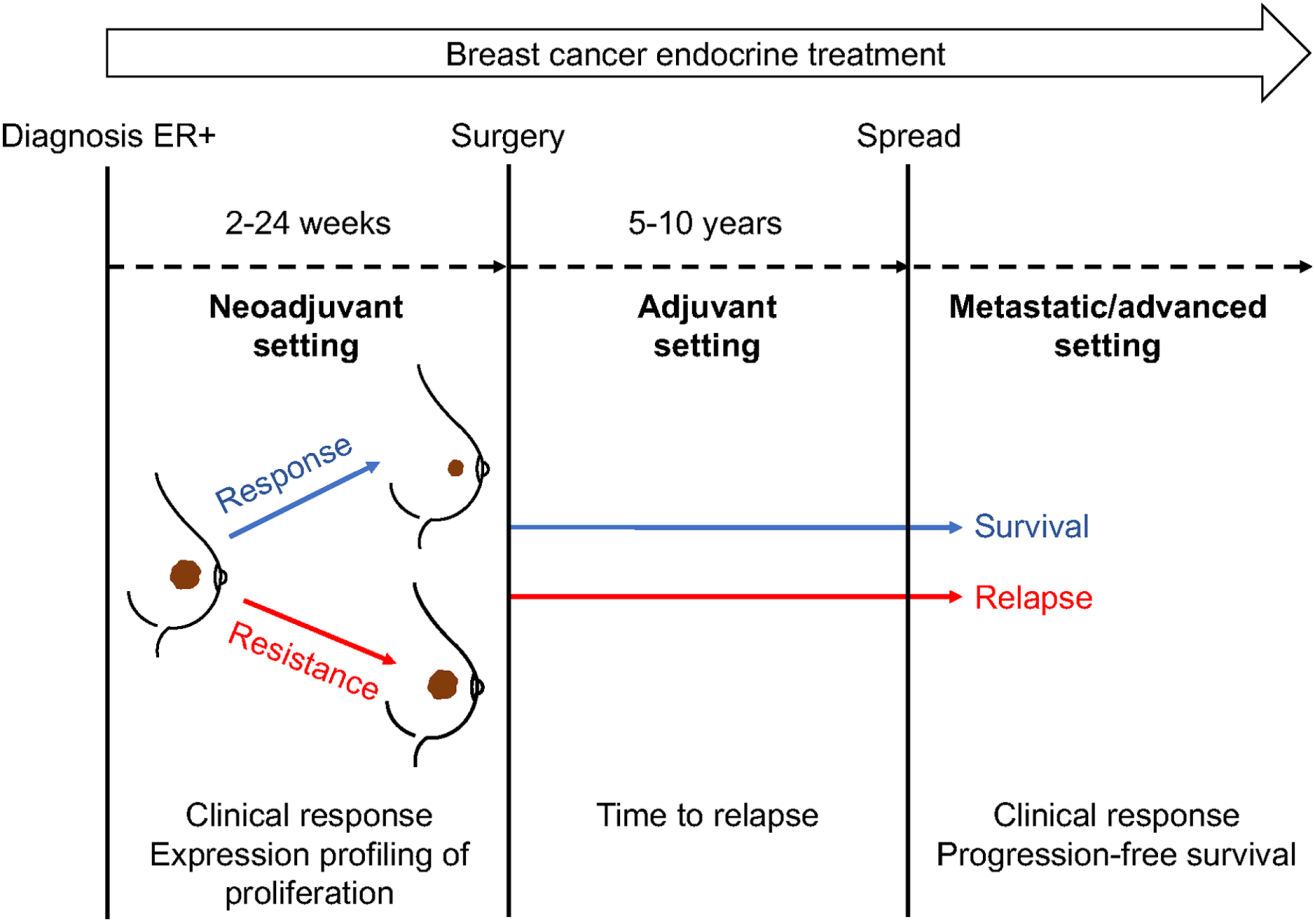
Main events and response endpoints over the course of endocrine treatment settings in breast cancer. Endocrine resistance is manifested clinically as an increase in tumour volume, relapse and progression in the neoadjuvant, adjuvant, and metastatic treatment setting, respectively. Biological endpoints have, also, been considered as an indication of endocrine treatment resistance. For instance, Ki67 is often measured either as a static marker of proliferation, or as a dynamic surrogate marker of drug response, when the expression levels are measured at multiple times during neoadjuvant treatment.

Despite the benefits from hormonal therapy, *de novo* and acquired resistance to treatment occur in a large number of patients and this significantly limits its optimal use in the clinic (Larionov & Miller 2009). Resistance is exhibited in different ways according to the disease/treatment setting (Fig. 1): (i) as disease recurrence during or after post-surgical adjuvant therapy; (ii) as a lack of tumour regression during neoadjuvant treatment, suggesting intrinsic resistance, or regrowth after an initial response, indicating acquired resistance; (iii) as progression of metastatic disease as (re-)growth of existing metastases or development of new metastases (Gonzalez-Angulo *et al.* 2007). Besides clinical response, the proliferation marker Ki67 has been used as a measure of response/resistance to neoadjuvant endocrine therapy (Selli & Sims 2019). Evidence from clinical trials of adjuvant endocrine therapy in the past suggested that disease recurred in up to 40–50% of ER-positive patients (Ring & Dowsett 2003, Dixon 2014), but contemporary rates are lower with more modern therapy used in patients with the earlier disease. In the neoadjuvant setting, clinical response rates range from 50 to 70% of patients (Colleoni & Montagna 2012). Almost all patients with advanced or metastatic ER+ breast cancer will relapse if treated with endocrine therapy alone during the first few years of treatment, and eventually die from the disease (D’Souza *et al.* 2018). The response can occur sequentially with different endocrine agents. The duration of response is increased by combination with other agents, such as cyclin-dependent kinase 4/6 (CDK4/6) inhibitors (Portman *et al.* 2019).

At diagnosis, around 15% of ER+ breast cancers exhibit a concurrent human epidermal growth factor receptor 2 (HER2) gene-amplification, such that approximately 10% of all breast cancers are ER+/HER2+ (Dodson *et al.* 2018). In most studies, ER+/HER2+ tumours have a more aggressive phenotype, as indicated by the patients’ poor prognosis and higher levels of tumour proliferation than those that do not demonstrate HER2-overexpression or gene amplification (Dowsett *et al.* 2001). Within HR+/HER2+ tumours, about 30% are considered HER2-enriched (HER2-E), the intrinsic subtype that is associated with high activation of the HER2 signalling pathway, enhanced proliferation, and increased numbers of tumour-infiltrating lymphocytes in the surrounding stroma and is characterised by a worse prognosis. Many tumours classified as HER2-E are not HER2+ by immunohistochemistry (IHC) or fluorescent *in situ* hybridisation (FISH) (Pascual *et al.* 2021). According to the American Society of Clinical Oncology (ASCO)/College of American Pathologists (CAP) updated guidelines on HER2 testing, HER2 IHC may be considered a screening test, and FISH can act as a confirmatory test for HER2 IHC equivocal cases. Thus, any IHC 3+ staining result indicates a HER2 positive diagnosis, and 0/1+ staining is considered negative. IHC 2+ results are considered positive if the FISH analysis indicates amplification with the updated criteria considering several scenarios based on HER2/CEP17 ratio and HER copy number (Wolff *et al.* 2018). Assigning HER2 status can also be complicated by the presence of intratumoural heterogeneity in HER2 overexpression, increase in chromosome enumeration probe 17 signals, alteration of HER2 status following neoadjuvant chemotherapy, or during metastatic progression (Ahn *et al.* 2020). These major nuances in HER2 status have rarely been addressed in studies of resistance to endocrine therapy and most likely contribute to different findings in different studies.

The degree of overexpression of HER2 is inversely correlated with ER expression (Ring & Dowsett 2003). This may relate to the repression of HER2 by ER through PAX2 and the ER coregulator AIB1/SRC3 competing for HER2 transcription (Hurtado *et al.* 2008). Increased HER2 protein expression due to expression loss of the oestrogen-regulated microRNA cluster comprising let-7c, miR99a, and miR125b, is another explanation of the inverse correlation between ER and HER2 (Bailey *et al.* 2015). This inverse correlation results in there being a greater proportion of HER2 immunohistochemistry (IHC) 2+ cases among ER+ than among ER− tumours.

**Figure 2:**
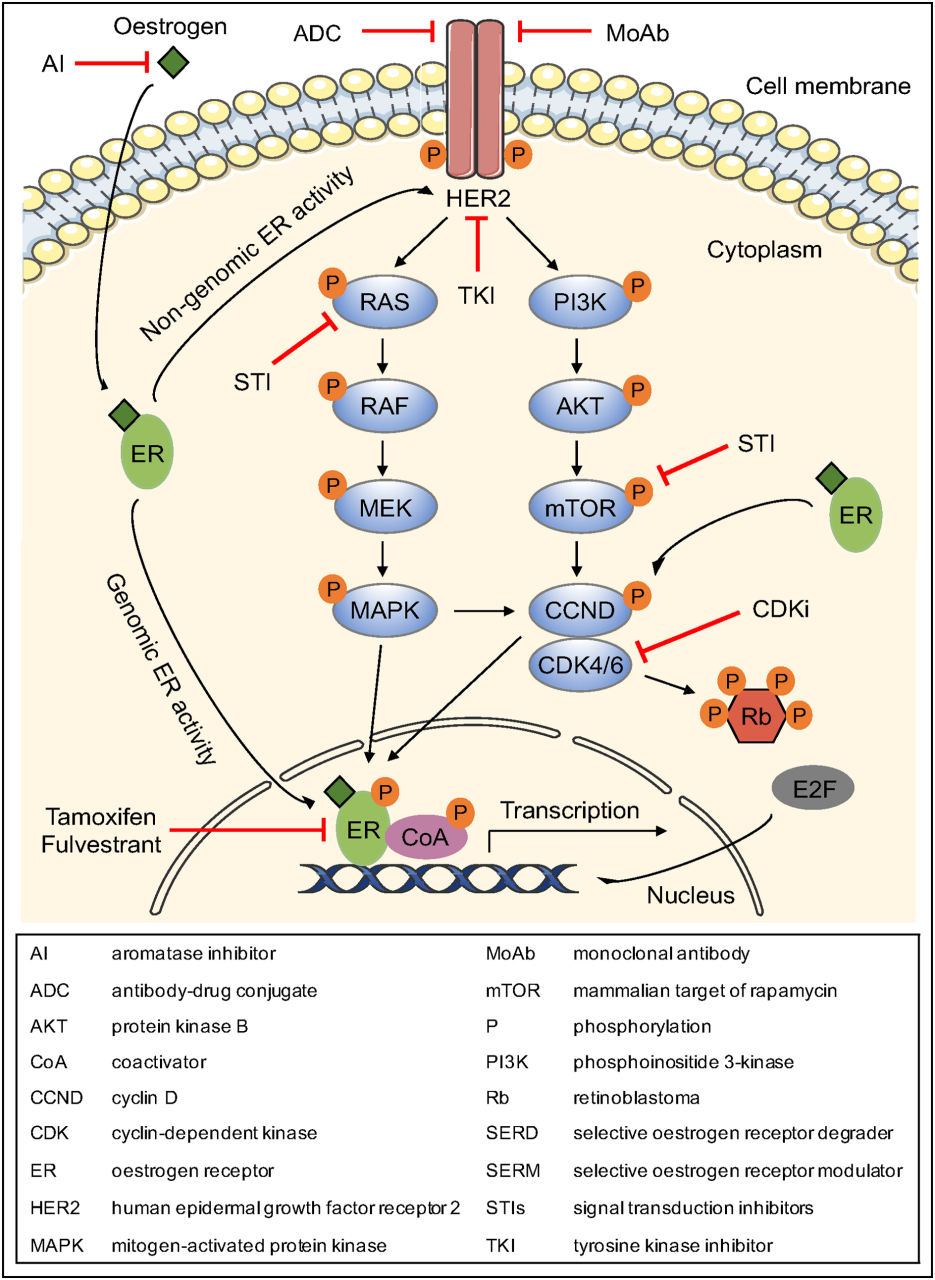
Crosstalk between ER and HER2 signalling pathways and its role in endocrine resistance and therapeutic agents that block specific molecules. A more detailed description is presented in the main text.

Compelling evidence suggests that breast cancer growth in at least some ER+ and HER2-overexpressing tumours is regulated by bi-directional crosstalk between ER and HER2 signalling pathways that can drive the development of resistance to endocrine therapy (Arpino *et al.* 2008, Goutsouliak *et al.* 2020) (Fig. 2). Several *in vitro* studies suggested that HER2 overexpression can facilitate both the genomic and non-genomic action of ER and its coactivator AIB1 in breast cancer cells, leading to tamoxifen resistance (Chung *et al.* 2002, Shou *et al.* 2004). Interestingly, upregulation of downstream signalling molecules, such as p42/44 mitogen-activated protein kinase (MAPK) and protein kinase B (AKT), is also an indication of endocrine resistance of breast cancer cells (Knowlden *et al.* 2003). It is plausible that HER2 overexpression might generate alternative signals of proliferation and survival to circumvent ER inhibition that subsequently result in endocrine therapy resistance.

In addition to HER2 protein overexpression or gene amplification, other predictive biomarkers have been reported for patients with HR+/HER2+ breast cancer, including gene expression scores, DNA mutations, proliferation, and the immune microenvironment (Dieci *et al.* 2020). Subtype classification of patients with metastatic HR+/HER2+ tumours using the PAM50 gene signature could be used as a useful tool for identifying patients with Luminal A tumours that exhibit longer progression-free survival (PFS) among other subtypes (Prat *et al.* 2016). In addition, *PIK3CA* and *ERBB2* mutations are associated with reduced pathological complete response rates and endocrine resistance (Loibl *et al.* 2016, Hyman *et al.* 2017). Another important predictor of resistance to endocrine therapies in HR+/HER2+ disease is the tumour immune microenvironment. Dunbier *et al.* have previously observed that an immune-gene signature was the strongest signature associated with poor antiproliferative response to an AI in a set of patients with ER+ tumours that were either HER2+ or HER2- (Dunbier *et al.* 2013).

Accumulating knowledge of the biology of this breast cancer subtype and understanding the mechanisms by which cells become resistant to endocrine therapies may provide useful information to refine the current treatment approach and enhance patients’ outcome. While there was much attention to the ER+/HER2+ subgroup of breast cancer in the previous decade, less attention has been paid in the recent past, most probably because of the advent of trastuzumab treatment and many other antibody-based or kinase-based anti-HER2 treatments. However, it is important to note that these anti-HER2 therapies are generally administered for a limited period; in the primary disease setting this is most often 12 months yet endocrine treatment is generally for at least 5 years and often for 10 years. Thus, any residual micro-metastatic ER+/HER2+ disease after surgery is not targeted by anti-HER2 therapy for the majority of the duration of endocrine treatment. In that case, recurrence may occur from persistent micro-metastatic disease that includes any remaining HER2+ clones.

The aim of this review is therefore to summarise the current knowledge on the response of patients with ER+ and HER2-overexpressing tumours to endocrine therapies comparing the different clinical treatment settings. This information is of paramount clinical importance, as it can provide a rational basis for the use of emerging combination therapies that may potentially evade endocrine resistance and eventually lead to complete tumour eradication.

## Role of HER2 in the development of endocrine resistance: *in vitro* and preclinical studies

Several preclinical studies suggested that growth factor signalling induces both *de novo* and acquired resistance of breast cancer cells to endocrine therapy. Overexpression of HER2 is associated with the development of *de novo* resistance of breast cancer cells to tamoxifen. For example, direct interaction between HER2 and ER in the BT474 HER2-overexpressing breast cancer model promoted resistance to tamoxifen by inhibiting its apoptotic effects (Chung *et al.* 2002). Benz *et al.* also demonstrated that xenograft tumours that were formed by inoculation of MCF7/HER2-18 cells stably transfected with HER2 were not sensitive to tamoxifen treatment (Benz *et al.* 1992). More recently, MCF7/HER2-18 cells were shown to be growth stimulated by tamoxifen in a low oestrogen environment, suggesting that tamoxifen can act as a potent agonist on tumour growth in this model (Shou *et al.* 2004).

In the above breast cancer cell lines, the presence of either oestrogen or tamoxifen instigates HER2 overexpression, which further enhances molecular crosstalk with the ER pathway (Shou *et al.* 2004). This leads to the activation of molecules involved in AKT and MAPK signalling pathways that, in turn, phosphorylate and augment the functional activity of ER and the coactivator AIB1, rendering breast cancer cells resistant to endocrine therapy (Shou *et al.* 2004, Arpino *et al.* 2008) (Fig. 2). Interestingly, although tamoxifen induces both genomic and non-genomic ER activation in HER2-overexpressing MCR7 cells *in vitro* (Chung *et al.* 2002, Shou *et al.* 2004), xenografts generated by the same cells *in vivo* are mainly characterised by non-genomic ER function as a mechanism of *de novo* resistance to tamoxifen (Massarweh *et al.* 2008). Moreover, tamoxifen induces the expression of oestrogen-regulated genes by facilitating the recruitment of coactivators, such as AIB1, rather than corepressor complexes in these HER2-overexpressing cells (Shou *et al.* 2004). The above events can be inhibited by treatment with anti-HER inhibitors, such as the EGF receptor (EGFR)/HER2 inhibitor, gefitinib, or the monoclonal anti-HER2 antibody, trastuzumab, which have the ability to eliminate the ER and HER2 crosstalk via blocking of EGFR/HER2 heterodimers or HER2, respectively, indicating that they are directly implicated in the growth-inducing role of tamoxifen in cells overexpressing HER2. In this regard, gefitinib or trastuzumab could restore tamoxifen’s anti-tumour effects in MCR7/HER2-18 cells, whereas it had only a modest effect on the inhibition of oestrogen-stimulated growth (Shou *et al.* 2004).

Experimental evidence suggested that acquired resistance to fulvestrant and tamoxifen can occur in continuous culture of breast cancer cells to these agents. Long-term culture of MCF7 cells with anti-oestrogens generated sublines that were insensitive and proliferated at rates equivalent to those of untreated WT cells (McClelland *et al.* 2001). The resistant MCF7 cells exhibited higher levels of EGFR and HER2 expression, enhanced activation of EGFR/HER2 heterodimers, and elevated levels of phosphorylation of MAPK, AKT, and nuclear ER (Knowlden *et al.* 2003). Similar to the *de novo* resistant models, gefitinib or trastuzumab, could act by hindering cell proliferation after acquiring resistance to tamoxifen (Knowlden *et al.* 2003). Taken together, the above *in vitro* and *in vivo* studies indicated that induced growth factor signalling and as a result, increased non-classic genomic or non-genomic ER activities, play an important role in the mechanism of both *de novo* and acquired resistance to anti-oestrogens. These mechanisms that sustain altered ER signalling in endocrine-resistant tumours can result in further unbalanced activity of ER co-regulator, ligand-independent ER activation, and altered ER-dependent transcriptional reprogramming to further support endocrine resistance (Massarweh *et al.* 2008, Evans *et al.* 2010, Lupien *et al.* 2010, Nardone *et al.* 2015).

## Role of HER2 in the development of endocrine resistance: clinical studies

### Endocrine therapy for metastatic disease

Conflicting results have been reported by several studies that assessed the effect of HER2 overexpression on endocrine resistance in the metastatic setting (Table 1) (Wright *et al.* 1992, Archer *et al.* 1995, Leitzel *et al.* 1995, Willsher *et al.* 1996, Yamauchi *et al.* 1997, Elledge *et al.* 1998, Houston *et al.* 1999, Hayes *et al.* 2001, Lipton *et al.* 2002, 2003). Some studies suggested that HER2 overexpression is associated with high levels of failure and poor response to endocrine treatment (Wright *et al.* 1992, Leitzel *et al.* 1995, Yamauchi *et al.* 1997, Houston *et al.* 1999, Hayes *et al.* 2001, Lipton *et al.* 2002, 2003), whilst others have not found enough evidence to verify this association (Archer *et al.* 1995, Willsher *et al.* 1996, Elledge *et al.* 1998).

**Table 1:** Summary of trials assessing HER2 in predicting responses to endocrine therapy for metastatic breast cancer.

Number of patients	HER2+ patients	HER2 assessment	Cut-off	Treatment	Endpoint	Response			Conclusion	Reference
						HER2+ patients	HER2− patients	*P*-value		
65	14 (22%)	IHC	≥50% of cells	Tamoxifen + ovarian ablation	OR (%)	20^b^	48^b^	*P* < 0.01	HER2 was significantly associated with poorer OR in tamoxifen-treated patients.	a
300^a^	58 (19.3%)	Serum ELISA	>30 U/mL	Megestrol acetate/ fadrozole	CR (%)	20.7	40.9	*P* = 0.004	HER2 was significantly associated with poorer response in patients treated with hormone therapies.	b
					CR (months)	11.6	15.5	*P* = 0.04		
					Trial survival (months)	15	28	*P* < 0.0001		
					Duration of cancer survival (months)	64.5	107.7	*P* < 0.0001		
92	24 (26%)	IHC	≥1% stained tumour cell membranes	Tamoxifen, goserelin, goserelin+tamoxifen, megestrol acetate	CR (%)	29.2	42.7	*P* = 0.24	HER2 was not significantly associated with poorer response in patients treated with hormone therapies.	c
52	13 (25%)	Serum ELISA	>20 ng/mL	Tamoxifen, tamoxifen+goserelin	CR (%)	50^b^	62.5^c^	*P* = 0.84	HER2 was not significantly associated with poorer response in patients treated with hormone therapies.	d
94	32 (34%)	Serum ELISA	≥5,000 U/mL	Droloxifene	OR (%)	9	56	*P* = 0.00001	HER2 was significantly associated with poorer OR in droloxifene-treated patients.	e
204^b^	61 (30%)	IHC	2+ (≥1% stained cells)	Tamoxifen	CR (%)	54	57	*P* = 0.67	HER2 was not significantly associated with poorer response, shorter TTF and OS in tamoxifen-treated patients.	f
					TTF (months)	6	8	*P* = 0.15		
					OS (months)	29	31	*P* = 0.36		
189^b^	57 (30.2%)	IHC	≥1% stained tumour cell membranes	Tamoxifen	Overall response (%)	24	64	*P* = 0.05	HER2 was significantly associated with shorter TTP in tamoxifen-treated patients.	g
					TTP (months)	5.5	11.2	*P* < 0.001		
103^a^	33 (32%)	Serum ELISA	>10.5 ng/mL	Megestrol acetate	OS (months)	20.2	27.8	*P* = 0.007	HER2 was significantly associated with shorter OS in patients treated with megestrol acetate.	h
					TTP (months)	6	7.4	*P* = 0.9		
					CR (%)	37	28	*P* = 0.41		
					CB (%)	80	78	*P* = 0.79		
711^a^	219 (30.8%)	Serum ELISA	>15 ng/mL	Fadrazole/letrozole/megestrol acetate	OR (%)	23	45	*P* < 0.0001	HER2 was significantly associated with poorer OS, and shorter TTP, TTF, response and OS in patients treated with hormone therapies.	i
					TTP (days)	90	180	*P* < 0.0001		
					TTF (days)	93	175	*P* < 0.0001		
					CR (months)	11.7	17.4	*P* < 0.0001		
					OS (months)	17.2	29.6	*P* < 0.0001		
562^b^	164 (29%)	Serum ELISA	>15 ng/mL	Tamoxifen/letrozole	OR (%)	15	32	*P* < 0.0001	HER2 was significantly associated with poorer response to tamoxifen and letrozole treatment.	j
					CB (%)	30	51	*P* < 0.0001		
					OR (months)	18.5	25.3	*P* < 0.014		
					CB (months)	16.3	20.9	*P* = 0.0067		
					TTP (months)	5.7	9.4	*P* < 0.0001		
					TTF (months)	4.2	9.1	*P* < 0.0001		
					OS (months)	20.8	36.5	*P* < 0.0001		

^a^ER-positive/ER-unknown, ^b^ER-positive, ^c^ER-negative; a (Wright *et al.* 1992), b (Leitzel *et al.* 1995), c (Archer *et al.* 1995), d (Willsher *et al.* 1996), e (Yamauchi *et al.* 1997), f (Elledge *et al.* 1998), g (Houston *et al.* 1999), h (Hayes *et al.* 2001), I (Lipton *et al.* 2002), j (Lipton *et al.* 2003).

CB, clinical benefit; CR, clinical response; ER, oestrogen receptor; HER2, human EGF receptor-2; IHC, immunohistochemistry; OR, objective response; OS, overall survival; TTF, time to treatment failure; TTP, time to progression.

This could be explained by the use of different techniques to evaluate HER2 status: determination of HER2 protein expression by IHC using a variety of antibodies (Wright *et al.* 1992, Archer *et al.* 1995, Elledge *et al.* 1998, Houston *et al.* 1999) or the assessment of serum circulating HER2 levels (Leitzel *et al.* 1995, Willsher *et al.* 1996, Yamauchi *et al.* 1997, Hayes *et al.* 2001, Lipton *et al.* 2002, 2003). With many different cut-off values being used to distinguish between HER2+ and HER2- status, it is hard to state precisely what percentage of patients overexpressed HER2. This issue was particularly relevant in the metastatic studies, which predated the publication of the ASCO/CAP guidelines for HER2 analysis and reporting (Wolff *et al.* 2007, 2018). For example, Elledge *et al*. suggested that inter-observer variability in IHC measurements could generate issues of reproducibility. For that reason, lack of a standardised scoring method for HER2 status could be accounted as a limitation (Elledge *et al.* 1998). Previous work has shown that only 30% of the HER2-overexpressing invasive human breast tumours were characterised by a phosphorylated form of the receptor, an indication of its active state (DiGiovanna *et al.* 1996). As the above methods used to define the HER2 status are not functional assays, it is also possible that the measured receptor protein is not activated.

In a number of studies, both ER+ and ER− tumours were included in the analysis (Wright *et al.* 1992, Archer *et al.* 1995, Hayes *et al.* 2001, Lipton *et al.* 2002, 2003), therefore much of the reported resistance of HER2-positive tumours to endocrine therapy could be due to the ER-negative nature of the tumour rather than HER2-positivity *per se* (Wright *et al.* 1992, Dowsett *et al.* 2001). When analysed separately, the number of tumours co-expressing ER and HER2 was very small in some studies (Wright *et al.* 1992, Willsher *et al.* 1996), such that results were underpowered and conclusions should be drawn with caution.

In studies undertaken in the metastatic setting, HER2 status has unavoidably often been assessed on the primary tumour, which differs temporally, topologically, and potentially biologically from the metastatic tumour (Dowsett *et al.* 2001). This makes it difficult to extrapolate any firm conclusion about the response of tumour in the metastatic sites. It is of particular note that approximately 20% of tumours, which are initially negative for HER2, can become positive over time as they progress, mainly following endocrine treatment (Gutierrez *et al.* 2005, Priedigkeit *et al.* 2017, 2021). This upregulation driven by ER blocking can be explained by the ability of ER to downregulate HER2 (Hurtado *et al.* 2008). In addition, *ERBB2* mutations have been acquired in ER+ metastatic breast cancer under the selective pressure of endocrine therapies resulting in treatment resistance (Razavi *et al.* 2018, Nayar *et al.* 2019, Bose & Ma 2021). A combination of endocrine therapies with the irreversible pan-HER tyrosine kinase inhibitor, neratinib, could overcome this resistance (Hyman *et al.* 2017, Nayar *et al.* 2019).

In an attempt to overcome the inconsistency between a number of the studies, De Laurentiis *et al.* conducted a meta-analysis of eight clinical trials including more than 1,900 patients with ER+ or ER-unknown disease to get an overall pooled estimate of the correlation between HER2 overexpression and the response to endocrine treatment. Overall, the pooled estimate of relative risk for all studies was 1.45 (95% CI, 1.34–1.57; *P* < 0.00001), indicating a significant association between HER2 overexpression and treatment failure. The test for heterogeneity (*P* = 0.27) showed that the differences among individual studies may be explained by chance and that combining data was an appropriate method. Interestingly, the interaction between HER2 overexpression and treatment failure was retained despite the endocrine therapy choice. The relative risk was 1.48 (95% CI, 1.29–1.70; *P* < 0.00001; test for heterogeneity = 0.09) for studies pertaining to tamoxifen, and 1.43 (95% CI, 1.30–1.58; *P* < 0.00001; test for heterogeneity = 0.64) for studies involving other endocrine drugs. The results suggested that patients with metastatic breast cancer and HER2 overexpression are relatively less responsive to endocrine therapies than patients in whom HER2 is not overexpressed. Importantly, however, because of the lack of a control arm, in which a therapeutic intervention was not administered, the more rapid progression may, at least to a certain degree, reflect a more aggressive inherent behaviour of the HER2-overexpressing tumours (De Laurentiis *et al.* 2005).

### Neoadjuvant endocrine therapy

The neoadjuvant setting presents the advantage that, unlike the adjuvant setting, the primary tumour is still in place as the treatment continues, and clinical efficacy outcomes can be captured by measurement of changes in tumour volume. Neoadjuvant studies are less time-consuming, and assessment of HER2 and ER status can directly be performed in the lesion being treated and in which response is measured (Ring & Dowsett 2003). Moreover, matched sequential tumour samples can be taken over time, allowing the study of dynamic biological changes during treatment (Dixon 2014). Characterising the molecular response to the treatment multiple times can be an important factor for a more accurate stratification of patients and subsequently for a more effective therapeutic decision making (Selli & Sims 2019). These data provide a unique opportunity to compare molecular and clinical determinants of early response and resistance throughout treatment as well as changes in molecular features that may determine endocrine responsiveness.

Because of the difference in pharmacological activity among types of endocrine therapy, it has been suggested in a neoadjuvant study that the choice of therapeutic agent might affect the responsiveness of HER2-overexpressing tumours (Ellis *et al.* 2001). Results of studies undertaken in the neoadjuvant setting are less disputable than those in the metastatic setting and offer firm evidence for the role of HER2 in tamoxifen resistance (Table 2). Overall, patients with ER+/HER2+ tumours have a poorer clinical response to tamoxifen than those with ER+/HER2− tumours, while they remain responsive to AIs (Ellis *et al.* 2001, 2003, 2006, Dixon *et al.* 2003, Zhu *et al.* 2004, Smith *et al.* 2005). Ellis *et al.* reported a study investigating the association between HER2 and EGFR protein expression by IHC, with response to tamoxifen over AIs. Patients, who were HR+ and overexpressing HER2 and/or EGFR, had a significantly greater clinical response to letrozole than to tamoxifen (88% vs 21%; *P* = 0.0004), while no significant difference was observed in ER+ and HER2− patients (54% vs 42%; *P* = 0.078). Moreover, rather than letrozole’s effects being diminished in HER2+ cases, letrozole was significantly more effective in patients overexpressing HER2 and/or EGFR compared to those that were negative for both receptors (88% vs 54%; *P* = 0.018) (Ellis *et al.* 2001). An update and expansion of the above study were presented by Ellis *et al.* in 2006 using FISH analysis to confirm HER2 status (Ellis *et al.* 2006). In contrast to the earlier report, letrozole was clinically effective in the 202 patients with ER+ tumours, irrespective of the HER2 status (71% in both subsets; *P* = 0.98), suggesting that they are sensitive to short-term oestrogen deprivation therapy. When ER+ tumours with HER2 gene amplification were treated with tamoxifen, the point estimate for the clinical response was poorer in HER2+ disease than in HER2− tumours, but this difference did not approach statistical significance (33% vs 49%; *P* = 0.49) (Ellis *et al.* 2006). Clinical findings of the Immediate Preoperative Anastrozole, Tamoxifen, or Combined with Tamoxifen (IMPACT) study suggested that HER2 overexpression was associated with poorer response in tamoxifen- but not in anastrozole-treated patients (22% vs 58%; *P* = 0.18).

**Table 2:** Summary of neoadjuvant trials investigating HER2 as a predictor of response to endocrine therapy for early breast cancer.

Number of patients	HER2+ patients	HER2 assessment	Cut-off	Treatment	Treatment duration	Endpoint	Response (%)					Conclusion	Reference
							HER2+ patients	HER2− patients	*P*-value				
									HER2+ vs HER2− comparison	Treatment comparison in HER2+	Treatment comparison in HER2−		
237^a^	36 (15.2%)^b^	IHC	2+/3+	Tamoxifen	4 months	CR	21	42	*P* = 0.095	*P* = 0.0004	*P* = 0.078	HER2 was associated with poorer CR in tamoxifen-treated patients, but not in letrozole-treated patients.	a
				Letrozole			88	54	*P* = 0.018				
22^a^	6 (27.3%)	IHC	3+	Anastrozole	4 months	CR	100	94	*P* > 0.05	NR	NR	HER2 was not associated with poorer CR in anastrozole-treated patients	b
185^a^	32 (17.3%)	IHC	2+/3+	Tamoxifen	4 months	CR	17	41	NR	*P* = 0.0002	*P* = 0.0534	HER2 was associated with poorer CR in tamoxifen-treated patients, but not in letrozole-treated patients.	c
				Letrozole			87	56	NR				
36^a^	16 (44.4%)	FISH	>2 HER2-to-chromosome 17 centromere ratio signals	Exemestane/letrozole	3 months	CR	75	35	*P* = 0.017	NR	NR	HER2 was associated with higher CR in AI-treated patients.	d
305^c^	28 (9.2%)	FISH	>2 HER2-to-chromosome 17 centromere ratio signals	Tamoxifen	4 months	CR	33	49	*P* = 0.49	*P* = 0.1	*P* = 0.0004	HER2 was associated with poorer CR in tamoxifen-treated patients, but not in letrozole-treated patients.	e
				Letrozole			71	71	*P* = 0.98				

Clinical response is referred to a reduction in tumour volume.

^a^HR-positive, ^b^HER2-positive or EGFR-positive, ^c^ER-positive; a (Ellis *et al.* 2001), b (Dixon *et al.* 2003), c (Ellis *et al.* 2003), d (Zhu *et al.* 2004), e (Ellis *et al.* 2006).

AI, aromatase inhibitor; CR, clinical response; EGFR, EGF receptor; ER, oestrogen receptor; FISH, fluorescent* in situ* hybridisation; HER2, human EGF receptor-2; HR, hormone receptor; IHC, immunohistochemistry; NR, not reported.

Besides clinical response, the effect of endocrine therapy on proliferation was used as a biological endpoint in neoadjuvant clinical studies (Table 3) (Dowsett *et al.* 2001, 2005*a*, Ellis *et al.* 2003, 2006, Dixon *et al.* 2004, Bliss *et al.* 2017). One of these studies showed that ER+/HER2+ tumours showed much less suppression of proliferation during AI treatment (Ki67 suppression: 45% vs 89.1%; *P* = 0.0001), even when a clinical response was observed (Ellis *et al.* 2006). In the IMPACT study, in which biological efficacy was assessed using the nuclear proliferation antigen Ki67, suppression of Ki67 was significantly greater in HER2− tumours compared to those overexpressing HER2 following either tamoxifen or anastrozole, but not the combination treatment (Dowsett *et al.* 2005*a*). The finding that tamoxifen-treated patients showed poorer clinical response could be explained by the reduced levels of apoptosis alongside Ki67 suppression. Interestingly, anastrozole still shrinks the tumour even when the levels of apoptosis are low (Dowsett *et al.* 2005*a*). These data suggest that clinical efficacy following endocrine treatment occurs following both decreased proliferation and maintained rate of apoptosis. Nevertheless, it is hard to study the effect of apoptosis in tumour regression following neoadjuvant endocrine treatment because only a few tumour cells are stained positive for apoptotic markers at baseline and they are not obviously modulated by the treatment (Dowsett *et al.* 2006). Despite the fact that the number of patients included in this analysis was small, the results agree with the findings of the letrozole study (Ellis *et al.* 2006). Although HER2 overexpression did not reduce the clinical benefit of neoadjuvant treatment with AIs, it was related to higher tumour proliferation before and during treatment than HER2− tumours.

**Table 3:** Clinical neoadjuvant studies of HER2 in predicting Ki67 changes in response to endocrine therapy.

Number of patients	HER2+ patients	HER2 assessment	Cut-off	Treatment	Endpoint	Response (%)			Conclusion	Reference
						HER2+ patients	HER2- patients	*P*-value		
115^a^	15 (13%)	IHC	2+/3+	SERM (tamoxifen/idoxifene) or AI (anastrozole/vorozole)	Ki67_B_	27.7	11.5	*P* = 0.003	HER2 was associated with high baseline Ki67 and small Ki67 change.	a
					Ki67_2w-B_	25	62	*P* = 0.014		
					Ki67_12w-B_	29	71	*P* = 0.047		
22^b^	6 (27.3%)	IHC	3+	Anastrozole	Ki67 reduction	67	80	*P* > 0.05	HER2 was not associated with smaller Ki67 changes in anastrozole-treated patients.	b
232^a^	34 (14.7%)	IHC	3+	Tamoxifen+anastrozole	Ki67_2w-B_	NR	NR	*P* < 0.05	HER2 was associated with smaller Ki67 changes in anastrozole- and tamoxifen-treated patients.	c
					Ki67_12w-B_	NR	NR	*P* < 0.05		
297^a^	26 (8.8%)	FISH	>2 HER2-to-chromosome 17 centromere ratio signals	Tamoxifen	Ki67 reduction	5.8	77.7	*P* = 0.0925	HER2 was associated with smaller Ki67 changes in tamoxifen- and letrozole-treated patients.	d
				Letrozole		45	89.1	*P* = 0.0001		
3861^a^	402 (10.4%)	IHC	3+	Anastrozole/letrozole	Ki67_B_	26.8	14.3	*P* < 0.001	HER2 was associated with high baseline Ki67 and small Ki67 change.	e
					Ki67_2w-B_	52.9	79.2	*P* < 0.001		

^a^ER-positive, ^b^HR-positive; a (Dowsett *et al.* 2001), b (Dixon *et al.* 2004), c (Dowsett *et al.* 2005), d (Ellis *et al.* 2006), e (Bliss *et al.* 2017).

AI, aromatase inhibitor; ER, oestrogen receptor; FISH, fluorescent *in situ* hybridisation; HER2, human EGF receptor-2; HR, hormone receptor; IHC, immunohistochemistry; Ki67_B_, Ki67 at baseline; Ki67_2w-B_, Ki67 reduction after 2 week-treatment; Ki67_12w-B_, Ki67 reduction after 12 week-treatment; NR, not reported; SERM, selective oestrogen receptor modulator.

### Endocrine therapy in the adjuvant setting

Several clinical studies have investigated whether HER2 protein overexpression or gene amplification influences the benefit of endocrine therapy in early-stage breast cancer in the adjuvant setting (Table 4) (Borg *et al.* 1994, Carlomagno *et al.* 1996, Berry *et al.* 2000, De Placido *et al.* 2003, Love *et al.* 2003, Dowsett *et al.* 2008, Rasmussen *et al.* 2008). Most of these studies suggested that early breast cancer patients with HER2-overexpressing tumours get less benefit from adjuvant tamoxifen than those with HER2− tumours and have an increased risk of failing tamoxifen treatment (Borg *et al.* 1994, Carlomagno *et al.* 1996, Hu & Mokbel 2001, De Placido *et al.* 2003, Dowsett *et al.* 2005*b*, 2008, Rasmussen *et al.* 2008). For example, an analysis of the Gruppo Universitario Napoletano 1 study concluded that tamoxifen was effective in reducing the hazard ratio of death among HER2− patients, while in contrast had rather a detrimental effect in ER+ patients with HER2-overexpressing tumours (0.73 vs 1.33, respectively; interaction test: *P* = 0.038) (De Placido *et al.* 2003). Nevertheless, only 91 of the 206 tamoxifen-treated patients were ER+ and/or PgR+ and only 58 had HER2 overexpression (De Placido *et al.* 2003), and the number of patients who were both ER+ and overexpressed HER2 was not reported in the paper, but it is likely to be low as a result of the inverse expression pattern of the two receptors (Ring & Dowsett 2003). As such, these findings should be interpreted critically.

**Table 4:** Clinical trials investigating the role of HER2 on the response to adjuvant endocrine therapy.

Number of patients	HER2+ patients	HER2 assessment	Cut-off	Treatment	Endpoint	Response (%)					Conclusion	Reference
						HER2+ patients	HER2− patients	*P*-value				
								HER2+ vs HER2- comparison	Treatment comparison in HER2+	Treatment comparison in HER2-		
871	175 (20.1%)	Southern blot	2–30 gene copies	Tamoxifen	OS	46	67	*P* < 0.0001	NR	NR	HER2 was associated with poorer OS in tamoxifen-treated patients.	a
		Western blot	100-3440 arbitrary units	Control		59	66	*P* = 0.34				
145	43 (29.7%)	IHC	≥10% of cells	Tamoxifen	OS	57	86	NR	*P* = 0.03	*P* = 0.09	HER2 was not associated with poorer DFS and OS in tamoxifen-treated patients.	b
				Control		82	68					
				Tamoxifen	DFS	51	82	NR	*P* = 0.3	*P* = 0.003		
				Control		63	54					
651^a^	12–24%^b^	IHC	≥50% of cells	Tamoxifen (compared to control)	OS (reduction in death)	30	36	NR	*P* = 0.18	*P* = 0.0037	HER2 was not associated with poorer DFS and OS in tamoxifen-treated patients.	c
		PCR	≥2 gene copies									
		FISH	≥2 HER2-to-chromosome 17 centromere ratio signals		DFS (reduction in RR)	32	39	NR	*P* = 0.12	*P* = 0.0001		
3533^a^	239 (6.8%)	FISH	≥2 HER2-to-chromosome 17 centromere ratio signals	Tamoxifen	DFS	70	86	NR	*P* < 0.0001	NR	HER2 was associated with poorer DFS in tamoxifen-treated patients. Letrozole improved DFS compared with tamoxifen regardless of HER2 status.	d
				Letrozole		79	90	*P* = 0.60				
1782^a^	187 (10.5%)	IHC	3+	Tamoxifen	Recurrence rate	18.8	9	*P* = 0.0018	NR	NR	HER2 was associated with shorter TTR in both tamoxifen- and anastrozole-treated patients.	e
				Anastrozole		19.8	5.9	*P* < 0.0001				

^a^ER-positive, ^b^depending on method used to assess HER2 status; a (Borg *et al.* 1994), b (Carlomagno *et al.* 1996), c (Berry *et al.* 2000), d (Rasmussen *et al.* 2008), e (Dowsett *et al.* 2008).

DFS, disease-free survival; ER, oestrogen receptor; FISH, fluorescent *in situ* hybridisation; HER2, human EGF receptor-2; IHC, immunohistochemistry; NR, not reported; OS, overall survival; PCR, PCR; RR, risk of recurrence; TTR, time to recurrence.

On the other hand, there are a few conflicting studies indicating no significant difference in disease-free and overall survival with adjuvant tamoxifen based on HER2 status (Berry *et al.* 2000, Love *et al.* 2003). One of these is the Cancer and Leukaemia Group B 8541 trial, which reported that patients with HER2+ disease, who received tamoxifen, had a 32 and 30% reduction in disease recurrence risk and death, respectively, compared to patients not receiving tamoxifen, a benefit not substantially less than the equivalent 39 and 36% seen in patients with HER2− disease. It should be noted though that the non-randomised nature of the study may have introduced a bias in the selection of patients towards a more advanced stage of tumour in the tamoxifen-treated group. Moreover, all patients received adjuvant chemotherapy, which might itself provide a benefit in the outcome of patients with HER2+ tumours (Berry *et al.* 2000).

More recent studies have investigated whether the outcome of patients, who received adjuvant AIs, differs according to HER2 status. The Breast International Group (BIG) 1-98 trial assessed the effect of HER2 status on the benefit of tamoxifen and letrozole in early breast cancer patients (Rasmussen *et al.* 2008). Letrozole improved disease-free survival (DFS) compared to tamoxifen irrespective of HER2 (HER2-negative: HR, 0.72; 95% CI, 0.59–0.87; HER2-positive: HR, 0.62; 95% CI, 0.37–1.03), thus, HER2 status should not be deemed as a selection criterion for letrozole over tamoxifen treatment (Rasmussen *et al.* 2008). Another study was conducted in order to determine the relationship of HER2 status with time to recurrence (TTR) in postmenopausal women with HR+ primary breast cancer in the large, randomised the Arimidex, Tamoxifen, alone or in combination (ATAC) adjuvant trial. Shorter TTR was observed in patients with HER2+ disease, who were treated with either anastrozole or tamoxifen. For anastrozole, the recurrence rate at 5 years was 19.8 and 5.9% for patients with HER2+ and HER2- tumours, respectively (HR, 2.25; *P* = 0.0018). For tamoxifen, it was 18.8 and 9% for patients with HER2+ and HER2- tumours, respectively (HR, 3.27; *P* < 0.0001). The benefit of anastrozole did not differ from that of tamoxifen in the HER2+ cohort, but there were only 44 patients in the HER2+ group (Dowsett *et al.* 2008). With varying treatment schedules and different techniques being used to score tumours as positive for HER2 expression, it is difficult to compare the two studies. Observations from the ATAC trial are in discordance with data from the IMPACT neoadjuvant trial, in which anastrozole did improve clinical benefit in HER2-overexpressing patients (Dowsett *et al.* 2008). Therefore, despite the initial effective tumour response to neoadjuvant AIs in the majority of patients with ER+/HER2+ disease, continued proliferation could hint early resistance that may occur at later times in the clinical course of the disease.

To provide higher-level evidence for the suggested association between HER2 status and benefit from endocrine therapy, a meta-analysis was conducted combining data from three randomised trials (ATAC, BIG 1-98, and TEAM). In patients with HER2- tumours, an improved outcome was observed following treatment with upfront AI compared to tamoxifen, while patients with HER2+ tumours showed no difference or slightly worse outcome in the first 2–3 years. Nevertheless, it should be noted that a large degree of heterogeneity in the HER2+ groups across all trials might be attributable to the small number of patients with HER2+ tumours, as well as subtle differences between AIs (Bartlett *et al.* 2017). Another meta-analysis of recurrence risk reductions combining data from five comparisons of AIs vs tamoxifen demonstrated that AI treatment was advantageous compared to tamoxifen regardless of HER2 status (EBCTCG 2015).

All these studies were limited in terms of assessing the markers and/or determinants of treatment resistance because of the absence of accessible disease after surgery. To produce meaningful and statistically valid results, adjuvant studies need a sufficiently large number of patient samples. The inverse correlation between ER and HER2 expression leads to there being only a minority population, generally around 10% of the total, in studies that recruited ER+ patients irrespective of HER2 status (Ring & Dowsett 2003). Direct comparison and interpretation of the cited studies are further hindered by the use of varying doses and durations of endocrine treatment, various assays and cut-off points for the assessment of ER and HER2 positivity, and confounders such as chemotherapy. Additionally, studies involving adjuvant treatment are made more difficult by the long-term follow-up required to assess disease endpoints and the occurrence or non-occurrence of an event being affected by the intrinsic prognosis of the disease as well as its response or not to therapy (Larionov & Miller 2009).

## Therapeutic strategies to combat endocrine resistance

Resistance of HR+/HER2+ breast cancer to endocrine therapy is heterogeneous and complex and may depend on the individual patient’s genetic background, the choice of endocrine therapy, and the type of resistance (Dixon 2014). There is convincing evidence that HER2 overexpression is a significant factor in endocrine resistance. However, stimulation of tumour growth is not solely the result of the crosstalk between ER and HER2, but rather the interaction of a more complex network (Arpino *et al.* 2008). Conceptually anti-HER2 therapies are given predominantly to target the drive that the tumour cells derive from overexpressed HER2. Such treatment may, however, antagonise HER2-dependent endocrine resistance directly as well as achieving a direct and independent effect on HER2-stimulated tumour growth. Understanding the underlying mechanisms would help to discover novel therapeutic agents and develop new strategies to overcome resistance. Historically, the majority of clinical trials assessing anti-HER2 therapies have not distinguished the ER status in HER2+ disease. Current guidelines recommend combination therapy of anti-HER2 agents including trastuzumab as the first-line treatment for HER2+ advanced breast cancer irrespective of HR status. Endocrine treatment could be limited to patients that are intolerant to chemotherapy or as an empirical maintenance strategy post-chemotherapy (Cardoso *et al.* 2020). Specifically, the use of tyrosine kinase inhibitors (e.g. lapatinib, tucatinib, neratinib, pyrotinib), monoclonal antibodies (e.g. trastuzumab, pertuzumab, margetuximab), chimeric antibodies with chemotherapeutic drugs, known as antibody-drug conjugates (e.g. trastuzumab emtansine (T-DM1), trastuzumab deruxtecan (DS-8201), trastuzumab duocarmazine (SYD985)) or other signal transduction inhibitors could halt the molecular interaction with the HER2 pathway (Mitsogianni *et al.* 2021) (Fig. 2). The ExteNET trial showed that 1-year neratinib treatment improved invasive DFS administered after chemotherapy and trastuzumab-based adjuvant therapy to patients with early-stage HER2+ breast cancer. Subgroup analysis demonstrated that neratinib gave greater benefit to patients with HR+ breast cancer than to those with HR− breast cancer, suggesting that there might be a preferential effect of TKIs on targeting the interaction of ER and HER2 than does the continuation of trastuzumab with endocrine treatment (Chan *et al.* 2016). It is also possible that the effect of TKIs could be dependent on a complete blockade of HER2 signalling and given the average lower expression of HER2 in ER+/HER2+ than ER+/HER2− disease this may be more easily achieved in the former.

Several studies exploring the use of growth factor receptor inhibitors in combination with anti-oestrogen therapy have shown improved outcomes for patients with either metastatic or locally advanced ER+/HER2+ disease (Johnston *et al.* 2009, Kaufman *et al.* 2009, Li *et al.* 2018, Rimawi *et al.* 2018). For example, data from the randomised open-label TAnDEM phase-III trial suggested that the addition of trastuzumab to anastrozole contributed to significant improvement in PFS in patients with ER+/HER2+ tumours compared to anastrozole treatment alone (5.6 months vs 3.8 months; *P* < 0.006) (Kaufman *et al.* 2009). This combination treatment, however, can be accompanied by adverse effects (Kaufman *et al.* 2009, Li *et al.* 2018), while in some cases it did not show higher efficacy in a subset of patients (Marcom *et al.* 2007, Burstein *et al.* 2014, Loi *et al.* 2016).

A secondary analysis of the HERA trial showed that a subgroup of patients with ER+/HER2+ breast cancer, with lower HER2 FISH ratios or higher *ESR1* mRNA expression, got less benefit from the addition of adjuvant trastuzumab following chemotherapy, with all these patients being given endocrine therapy. This observation indicates that the degree of *HER2* amplification and *ESR1* expression levels can affect the response to trastuzumab after chemotherapy in the ER+/HER2+ disease and may explain the heterogeneity in response to anti-HER2 agents in this subgroup (Loi *et al.* 2016). The lower amount of benefit seen in patients with high ER-expressing tumours is consistent with the observation in the recently published overview of trastuzumab trials in which ER+/PgR+ cases appear to receive less benefit than ER+/PgR− cases in which ER levels are lower and HER2 levels are higher (Arpino *et al.* 2005, EBCTCG 2021). Furthermore, AIB1 overexpression has been associated with tamoxifen resistance in hormone-responsive HER2+ breast cancers, suggesting that AIB1 could be a potential therapeutic target (Osborne *et al.* 2003, Kirkegaard *et al.* 2007).

Potential additional benefit of the utilisation of other therapeutic approaches is currently undergoing clinical investigation, such as the synergistic combination of endocrine therapy plus dual HER2-targeted therapy and a CDK4/6 inhibitor in patients with ER+/HER2+ metastatic disease (Krause *et al.* 2019). Recently, data from the prospective, open-label, multicentre phase-II SOLTI-1303 PATRICIA trial showed that palbociclib in combination with trastuzumab is safe and results in longer PFS in trastuzumab pre-treated ER+/HER2+ advanced breast cancer with a PAM50 luminal subtype (12.4 months vs 4.1 months; *P* = 0.025) (Ciruelos *et al.* 2020). A number of studies focussed on investigating the treatment of patients with ER+/HER2-low (HER2 IHC 1+/2+, FISH negative) breast cancer, which is not effectively treated with first-generation anti-HER2 agents, such as trastuzumab. The addition of zenocutuzumab (MCLA-128) to endocrine therapy in ER+/HER2-low metastatic breast cancer patients, who had progressed on endocrine therapy and CDK4/6 inhibitors, resulted in a rescue of endocrine sensitivity of 17% (Pistilli *et al.* 2020). The combination of DS-8201 with anastrozole or fulvestrant is currently being assessed in patients with ER+/HER2-low metastatic breast cancer in the DESTINY-Breast08 phase Ib trial (Jhaveri *et al.* 2021).

## Conclusion

A number of clinical trials have investigated the role of HER2 on the resistance to endocrine therapy over the last decades. In some cases, the results from these studies should be interpreted with caution because of the limited number of patients with ER and HER2 co-expression, the different endocrine therapies administered, and the range of techniques used for the detection of HER2 expression. However, data have given useful prognostic information in the neoadjuvant, adjuvant, and metastatic settings. Metastatic ER+/HER2+ breast cancers appear to be less responsive to both tamoxifen and AIs than ER+/HER2− disease. Evidence from adjuvant and neoadjuvant studies supports the idea that patients with ER+/HER2+ disease may benefit from AIs more than tamoxifen in the same way as ER+/HER2− tumours. Clinical studies suggest that continued proliferation even in the presence of clinical response following neoadjuvant AI treatment could indicate resistance that could be developed at a later stage. The sensitivity of biological endpoints highlights the importance of introducing them into clinical practice, which alongside clinical outcome, could portend a better prognosis. Selection of biomarkers, such as Ki67, to stratify patients into clinically distinctive groups in a move towards personalised therapy will aid to improve poor responsiveness to anti-oestrogen therapies in patients with ER+/HER2+ tumours.

In the future, analyses of circulating tumour DNA may enable the tracking of the response of subclinical disease to anti-HER2 with or without anti-ER treatment, and thereby aid both clinical management of patients and provide improved data for understanding the interaction of ER and HER2 signalling in patients. Other potential biomarkers, such as gene expression signatures, DNA mutations, and the tumour immune microenvironment, could predict the responsiveness of patients with ER+/HER2+ disease to endocrine therapies (Dieci *et al.* 2020). Determining the most suitable way to monitor response is thus of paramount importance.

The nature of anti-oestrogen therapy resistance in patients with HER2 overexpressing tumours is relative rather than absolute, therefore this therapeutic option should not necessarily be withheld. Ongoing and future clinical trials will evaluate the potential and applicability of combining endocrine therapy with growth factor inhibitors or CDK4/6 inhibitors to overcome intrinsic resistance and prevent or delay acquired resistance in patients with ER+/HER2+ breast cancer.

## Declaration of interest

A A has no competing interests. M D served on advisory boards in Radius, G1 therapeutics, AbbVie, Zentalis, H3 Biomedicine; received lecture fees from Nanostring, Myriad, Lilly.

## Funding

This work was supported by the National Institute for Health Research (NIHR) Biomedical Research Centre at the Royal Marsden NHS Foundation Trust and The Institute of Cancer Research, London. We also thank Breast Cancer Now for funding this work as part of Programme Funding to the Breast Cancer Now Toby Robins Research Centre. A A was supported by a grant from Le Cure Breast Cancer Research Fund. The funding bodies did not have a role in the design, analysis or interpretation of this review.

## Availability of data and materials

Not applicable as no datasets were generated during this study.

## Ethics approval and consent to participate

Not applicable as this review does not contain any studies with human patients or animals performed by any of the authors.

## Author contribution statement

A A searched and evaluated all relevant literature and wrote the manuscript. M D conceived the topic idea, supervised the search strategy, and reviewed and revised the manuscript drafts. All authors read and approved the final manuscript.
